# CircST6GAL1 knockdown alleviates pulmonary arterial hypertension by regulating miR‐509‐5p/multiple C2 and transmembrane domain containing 2 axis

**DOI:** 10.1111/crj.13771

**Published:** 2024-05-15

**Authors:** Xing Zhang, Hao Qin, Qiang Ma, Junbo Zhang, Hongyan Tian, Yan Meng

**Affiliations:** ^1^ Department of Peripheral Vascular Diseases The First Affiliated Hospital of Xi'an Jiaotong University Xi'an China

**Keywords:** circST6GAL1, HPASMCs, MCTP2, miR‐509‐5p, pulmonary arterial hypertension

## Abstract

**Background:**

Hypertension is a main contributing factor of cardiovascular diseases; deregulated circular RNAs are involved in the pathogenesis of pulmonary arterial hypertension (PAH). Herein, we evaluated the function and mechanism of circST6GAL1 in PAH process.

**Methods:**

Human pulmonary artery smooth muscle cells (HPASMCs) were cultured in hypoxic environment for functional analysis. The cell counting kit‐8, 5‐ethynyl‐2′‐deoxyuridine, wound healing, and flow cytometry assays were used to investigate cell proliferation, migration, and apoptosis. qRT‐PCR and Western blotting analyses were used for level measurement of genes and proteins. The binding between miR‐509‐5p and circST6GAL1 or multiple C2 and transmembrane domain containing 2 (MCTP2) was analyzed by dual‐luciferase reporter, RNA immunoprecipitation, and pull‐down assays. The monocrotaline (MCT)‐induced PAH mouse models were established for in vivo assay.

**Results:**

CircST6GAL1 was highly expressed in PAH patients and hypoxia‐induced HPASMCs. Functionally, circST6GAL1 deficiency reversed hypoxia‐induced proliferation and migration, as well as apoptosis arrest in HPASMCs. Mechanistically, circST6GAL1 directly targeted miR‐509‐5p, and MCTP2 was a target of miR‐509‐5p. Rescue assays showed that the regulatory effects of circST6GAL1 deficiency on hypoxia‐induced HPASMCs were abolished. Moreover, forced expression of miR‐509‐5p suppressed HPASMC proliferation and migration and induced cell apoptosis under hypoxia stimulation, while these effects were abolished by MCTP2 overexpression. Moreover, circST6GAL1 silencing improved MCT‐induced pulmonary vascular remodeling and PAH.

**Conclusion:**

CircST6GAL1 deficiency reversed hypoxia‐induced proliferation and migration, as well as apoptosis arrest in HPASMCs, and alleviated pulmonary vascular remodeling in MCT‐induced PAH mouse models through the miR‐509‐5p/MCTP2 axis, indicating a potential therapeutic target for PAH.

## Graphical abstract

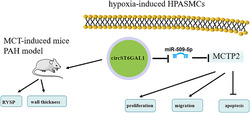

## 1 INTRODUCTION

Pulmonary arterial hypertension (PAH) is a chronic progressive disorder affecting lung arteries and right ventricular. It is featured by angioproliferative vasculopathy and structural remodeling in pulmonary arteries, resulting in uncontrolled proliferation and dysfunction of smooth muscle cells (SMCs).^1^ This vasculopathy increases the resistance of pulmonary vascular and succeeding pulmonary artery pressure, leading to right ventricular failure or even death.^2^, ^3^ PAH is still tightly related to significant morbidity and mortality.^4^ Therefore, further investigations on the pathogenesis of PAH might aid in increasing the repertoire of drugs available.

Circular RNAs (circRNAs) show high stability due to their covalently closed loop.^5^ Functionally, circRNAs have function in modulating diverse physiological processes in cells.^6^, ^7^, ^8^ Moreover, deregulated circRNAs are associated with the malignant progression of cardiovascular diseases. For example, circ_0091822 enhanced ox‐LDL‐induced migration, invasion, and proliferation in vascular SMCs (VSMCs) during atherosclerosis.^9^ Deficiency of circItgb5 is alleviated in PAH rat model.^10^ CircRNA FEACR could suppress ferroptosis and ameliorated myocardial ischemia/reperfusion injury.^11^ CircST6GAL1 (has_circ_0068481) is originated from ST6GAL1 gene in chr3: 186756529‐186761098; it was found to be elevated in the serum of idiopathic PAH patients and might be a sensitive and specific marker for idiopathic PAH prediction.^12^ Here, we speculated that circST6GAL1 might participate in the process of PAH.

As epigenetic modulators, circRNAs can act as sponges for microRNAs (miRNAs/miRs) to sequester miRNAs and modulate downstream mRNAs.^13^, ^14^ Herein, the miRNA/mRNA axis was predicted using online bioinformatics software, and it was found that miR‐509‐5p has complementary sequences on circST6GAL1 and multiple C2 and transmembrane domain containing 2 (MCTP2). Hypoxia can affect the pulmonary circulation and induce pulmonary vasoconstriction and vascular remodeling in the lungs, ultimately increasing pulmonary artery pressures.^15^, ^16^ Hence, this study used hypoxia‐induced human pulmonary artery SMCs (HPASMCs) in vitro and established monocrotaline (MCT)‐induced PAH mice in vivo to investigate the action of circST6GAL1 on PAH and further explored whether circST6GAL1 functioned by regulating miR‐509‐5p and MCTP2.

## 2 MATERIALS AND METHODS

### 2.1 Clinical samples collection

The blood samples were collected from 37 PAH patients and 17 age‐ and sex‐matched healthy individuals without medications or medical conditions (control) at the First Affiliated Hospital of Xi'an Jiaotong University. The serum was obtained by centrifugation, and all samples were kept at −80°C until further studies. This study was authorized by the Ethics Committee of the First Affiliated Hospital of Xi'an Jiaotong University and was carried out according to the guidelines of Declaration of Helsinki, and the written informed consents from all subjects had been collected.

### 2.2 Cell culture and treatment

HPASMCs were purchased from Shanghai Institutes for Biological Sciences (Shanghai, China) and cultured in DMEM plus 10% FBS and 1% penicillin–streptomycin (all from Thermo Fisher Scientific, Waltham, MA, USA). In functional experiments, cells were cultivated in a hypoxic environment with 5% CO_2_ and 3% O_2_ at 37°C (hypoxia group). In the normoxia group, cells were maintained in an incubator with 5% CO_2_ and 21% O_2_ at 37°C.

### 2.3 qRT‐PCR

The cytoplasmic and nuclear RNAs were isolated using the PARIS kit (Thermo Fisher Scientific) under recommended condition. Total RNAs were isolated using TRIzol reagent (Takara, Dalian, China). Then, cDNAs were generated using PrimeScript RT Reagent Kit, and qRT‐PCR was carried out by TB Green Premix Ex Taq II (Takara) with β‐actin or U6 as the internal control. Table 1 showed the primers for qRT‐PCR.

**TABLE 1:** Primers sequences used for qRT‐PCR.

Name		Primers for qRT‐PCR (5′‐3′)
CircST6GAL1	Forward	AGCATTAGGACCAAGGCTGG
	Reverse	TGATCAAAACTCATCGATTTC
ST6GAL1	Forward	CCCCTCTTTCGAGACTCCCT
	Reverse	GTCGGTATCGGCTCCCTTTG
MCTP2	Forward	GCCTCGCACAGGAGTCATTG
	Reverse	GATCTGGGGGCTTACTTGGG
miR‐509‐5p	Forward	GTATGATACTGCAGACAGTGG
	Reverse	CTCAACTGGTGTCGTGGAG
β‐actin	Forward	GACTCCAAGGCCACGGATAG
	Reverse	TGTTCGAGGATCTGTGCCAA
U6	Forward	CTCGCTTCGGCAGCACA
	Reverse	AACGCTTCACGAATTTGCGT

### 2.4 RNase R assay and actinomycin D treatment

About 2 μg of total RNAs was incubated with 3 U/μg of RNase R or not for 30 min at 37°C. The resulting RNAs were collected and analyzed by qRT‐PCR.

To block transcription, HPASMCs were treated with 2 μg/mL actinomycin D (Abcam, Cambridge, MA, USA) in 24‐well plates, and then, qRT‐PCR was adopted to test circST6GAL1 and ST6GAL1 mRNA expression levels.

### 2.5 Cell transfection

CircST6GAL1‐specific small interference RNAs (si‐circST6GAL1), pCD5‐ciR‐circST6GAL1 overexpression plasmids, pcDNA3.1‐MCTP2 overexpression plasmids, and the contrasts (si‐con, pCD5‐ciR, or pcDNA) were synthesized by Genema (Shanghai, China). The miR‐509‐5p mimic or inhibitor (miR‐509‐5p or in‐miR‐509‐5p) and the control (miR‐con or in‐miR‐con) were provided by RiboBio (Guangdong, China). The transfection was conducted adopting Lipofectamine 2000 (Thermo Fisher Scientific) based on the experimental design. After confirming the transfection efficiency, cells were stimulated by hypoxia condition.

### 2.6 Cell counting kit‐8 assay

HPASMCs (2 × 10^3^) were inoculated into a 96‐well plate for 0, 24, 48, or 72 h. Each well was added with 10 μL of cell counting kit‐8 solution (Beyotime, Shanghai, China) at indicated times. Four hours later, the absorbance values of the cells were detected at 450 nm.

### 2.7 5‐Ethynyl‐2′‐deoxyuridine (EdU) assay

HPASMCs (5 × 10^3^ cells per well) were incubated with 50 μM EdU labeling solution (RiboBio) in a 96‐well plate for 3 h. After being fixed with 4% formaldehyde, cells were stained with 1× Apollo® reaction cocktail for a half hour, followed by dyeing with DAPI. Next, EdU‐positive cells were photographed and calculated.

### 2.8 Wound healing assay

HPASMCs were seeded into the eight‐well plate with DMEM and 10 μg/mL mitomycin C. When the cell confluence reached approximately 95%, the wound was made by a sterile pipette tip; after washing, the images were captured (0 h). Following 24 h incubation, the wound distance was photographed (24 h), and wound closure was analyzed by ImageJ software to calculate the migration rate.

### 2.9 Flow cytometry

HPASMCs were resuspended in 500 μL binding buffer (3 × 10^5^ cells per mL), followed by dyeing with 5 μL Annexin V‐FITC and propodium iodide (5 μL) (BestBio, Shanghai, China) away from light. Fifteen minutes later, cell apoptosis was assessed by the flow cytometer.

### 2.10 Western blotting

Total protein was extracted by incubating with RIPA lysis buffer (Beyotime) for 30 min, and the BCA method was adopted to determine protein concentrations. About 30 μg proteins were fractionated on 10% SDS‐PAGE gel and shifted onto nitrocellulose membranes. The membranes were then incubated at 4°C all night with BAX (1:1000, ab32503), BCL‐2 (1:1000, ab692), MMP2 (1:500, ab37150), PCNA (1:5000, ab29), and β‐actin (1:1000, Abcam), followed by incubating with incubated secondary antibodies for 2 h at 37°C. The blot signal was analyzed by ECL substrate (Beyotime).

### 2.11 Dual‐luciferase reporter assay

The binding site between miR‐509‐5p and circST6GAL1 or MCTP2 was predicted by online bioinformatics software (Circbank or microT_CDS). The wild‐type circST6GAL1 or MCTP2 (circST6GAL1‐WT or MCTP2‐WT) and the mutated circST6GAL1 or MCTP2 (circST6GAL1‐MUT or MCTP2‐MUT) were provided by RiboBio and then inserted into the psiCHECK‐2 vector (Promega, Beijing, China). Then, HPASMCs were transfected with 200 ng recombinant vectors and 50 nM miR‐509‐5p or miR‐con; 48 h later, firefly and renilla luciferase activities were assayed.

### 2.12 RNA immunoprecipitation (RIP) assay

The magnetic beads were preincubated with antibodies specific for Ago2 or IgG (all from Millipore, Billerica, MA, USA). HPASMCs were lysed and incubated with the antibody‐coated beads. Finally, coprecipitated RNAs were isolated for qRT‐PCR analysis.

### 2.13 Pull‐down assay

Biotinylated miR‐509‐5p (bio‐miR‐509‐5p‐WT) or the mutant (bio‐miR‐509‐5p‐MUT) and the control (bio‐miR‐con) were synthesized by RiboBio and transfected into HPASMCs for 48 h incubation. Then, cells were lysed and incubated with streptavidin‐coated magnetic beads. Lastly, RNA complex was purified by TRIzol, and the abundance of circST6GAL1 or MCTP2 was detected.

### 2.14 Animal studies

Short hairpin RNAs targeted circST6GAL1 (sh‐circST6GAL1) and a scrambled short hairpin RNA (sh‐NC) were designed, and AAV9‐sh‐circST6GAL1 or AAV9‐sh‐NC virus was constructed by BrainVTA (Wuhan, China). In total, 24 male C57/BL6 mice (SPF, 4–5 weeks old, 25–30 g, Slaike Jingda Laboratory, Hunan, China) were included in this study and divided into four groups: Control, MCT, MCT+ sh‐NC, or MCT+ sh‐circST6GAL1 group. The animal study was approved by the Ethics Committee of the First Affiliated Hospital of Xi'an Jiaotong University. Animal studies were performed in compliance with the ARRIVE guidelines and the Basel Declaration. Mice in MCT group were subcutaneously injected with 60 mg/kg MCT (Sigma‐Aldrich, St. Louis, MO). Mice in control group received an equivalent volume of saline. MCT+ sh‐circST6GAL1 or MCT+ sh‐NC mice were injected AAV9‐sh‐circST6GAL1 or AAV9‐sh‐NC 10 days before MCT injection via the tail vein. Right ventricular systolic pressure (RVSP) was determined after MCT treatment in mice as described in previous reports.^10^, ^17^ After 30 days, all the mice were anesthetized with isoflurane and killed, the pulmonary artery specimens were collected, and part of the tissues was stored in 4% paraformaldehyde for hematoxylin and eosin staining and immunohistochemistry analysis, and the remaining tissues were frozen in liquid nitrogen for Western blotting.

### 2.15 Statistical analysis

The data were manifested as the mean ± standard deviation. The correlation between groups was assessed with Pearson correlation coefficient. Differences between groups were conducted using ANOVA followed by Tukey's post‐test or Student's *t*‐test. *P < 0.05 suggested statistically significant.

## 3 RESULTS

### 3.1 CircST6GAL1 is highly expressed in PAH and hypoxia‐induced HPASMCs

**FIGURE 1:**
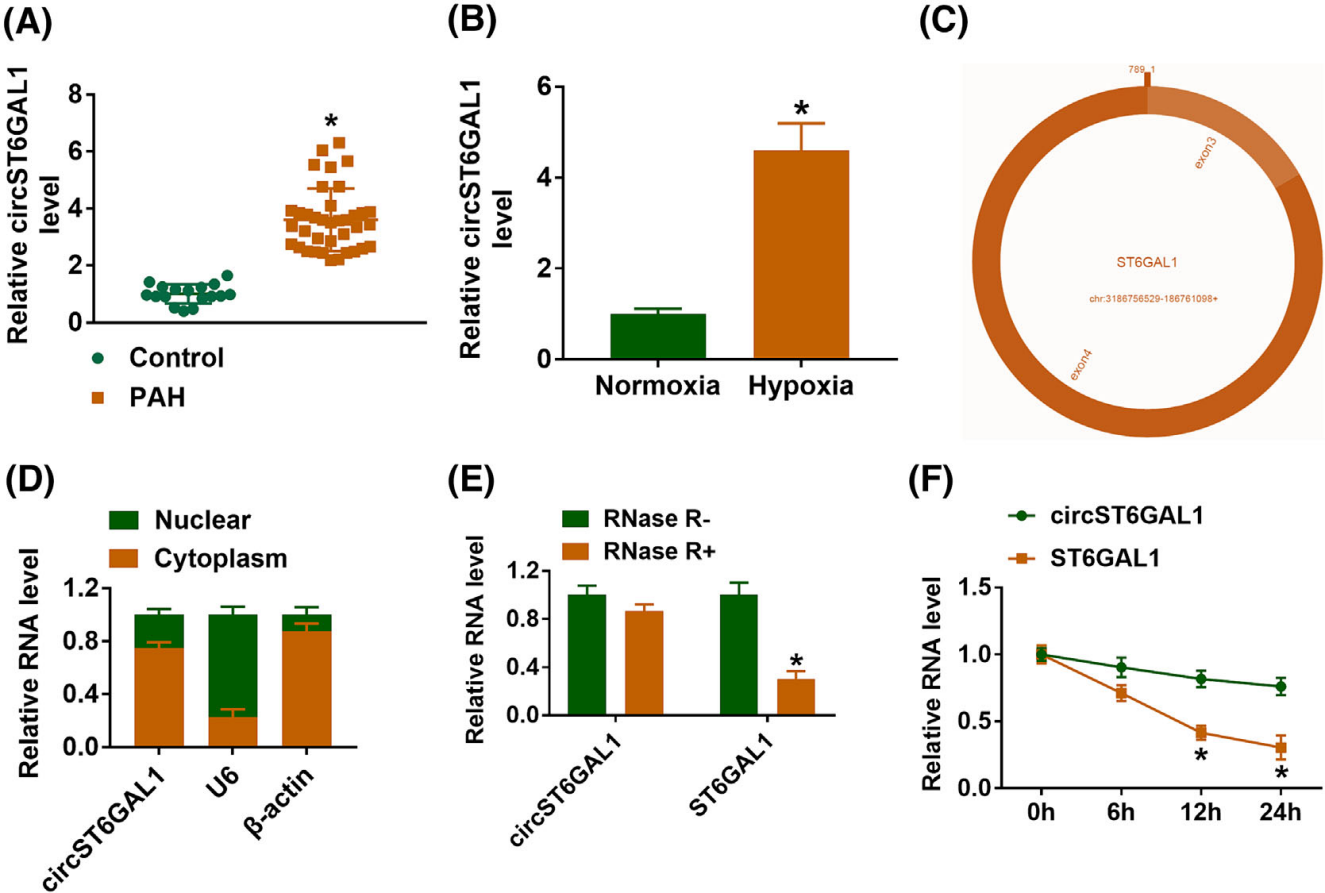
CircST6GAL1 is highly expressed in pulmonary arterial hypertension (PAH) and hypoxia‐induced human pulmonary artery smooth muscle cells (HPASMCs). (A, B) qRT‐PCR analysis for the expression of circST6GAL1 in PAH patients or healthy individuals, as well as in hypoxia‐ or normoxia‐induced HPASMCs. (C) Schematic illustration showing the genomic location of circST6GAL1 generated from ST6GAL1 gene. (D) qRT‐PCR suggesting the distribution of circST6GAL1 in the cytoplasmic and nuclear fractions of HPASMCs. (E, F) The stability of circST6GAL1 was determined using RNase R or actinomycin D, respectively. *P < 0.05.

Compared with the healthy control, circST6GAL1 expression was higher in the serum of PAH patients (Figure 1A). Also, its expression was increased in hypoxia‐induced HPASMCs relative to normoxia‐treated cells (Figure 1B). Therefore, circST6GAL1 deregulation might be associated with PAH. CircST6GAL1 was looped and comprised exons 3 to 4 of ST6GAL1 gene (Figure 1C). It was found to be mainly distributed in the cytoplasmic fractions of HPASMCs (Figure 1D), suggesting that circST6GAL1 may exert regulatory effects via acting miRNA sponges. In addition, circST6GAL1 was resistant to the digestion by RNase R or actinomycin D (Figure 1E,F), indicating that circST6GAL1 is a stable circRNA.

### 3.2 CircST6GAL1 knockdown reverses hypoxia‐induced HPASMC dysfunction

**FIGURE 2:**
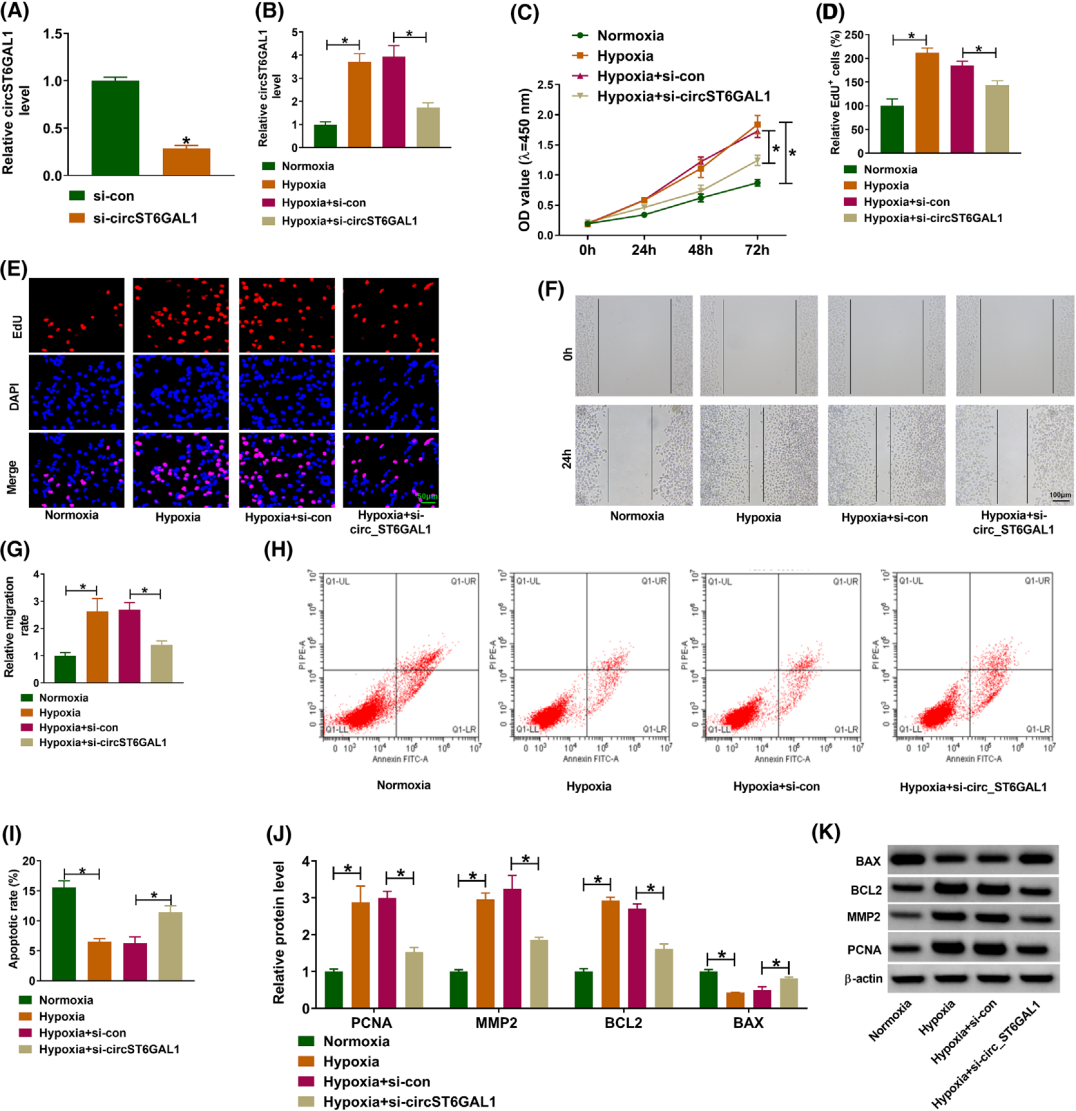
CircST6GAL1 knockdown reverses hypoxia‐induced human pulmonary artery smooth muscle cell dysfunction. (A) The interference efficiency of si‐circST6GAL1 or si‐con was validated using qRT‐PCR. (B–K) Human pulmonary artery smooth muscle cells were transfected with si‐circST6GAL1 or si‐con and then incubated in hypoxia condition. (B) qRT‐PCR for circST6GAL1 expression. (C–E) Cell counting kit‐8 and 5‐ethynyl‐2′‐deoxyuridine (EdU) assays for proliferation analysis. (F, G) Wound healing assay for cell migration. (H, I) Flow cytometry for cell apoptosis. (J, K) Western blotting analysis for the protein levels of PCNA, MMP2, BCL2, and BAX. *P < 0.05.

Next, the role of circST6GAL1 was analyzed. CircST6GAL1 siRNA (si‐circST6GAL1) was designed; as expected, si‐circST6GAL1 transfection markedly decreased circST6GAL1 expression in HPASMCs compared with si‐con transfection (Figure 2A). Then, HPASMCs were transfected with si‐circST6GAL1 or si‐con and then incubated in hypoxia condition. qRT‐PCR analysis showed that hypoxia‐induced circST6GAL1 increase was reduced by si‐circST6GAL1 in HPASMCs (Figure 2B). Functionally, it was confirmed that hypoxia promoted the proliferation (Figure 2C–E) and migration (Figure 2F,G) of HPASMCs, while these effects were reversed after circST6GAL1 silencing. In addition, the apoptosis was suppressed by hypoxia and then rescued by si‐circST6GAL1 in HPASMCs (Figure 2H,I). Moreover, the related markers of proliferation, migration, and apoptosis were analyzed. As shown in Figure 2J,K, the levels of PCNA, MMP2, and BCL‐2 were increased, and BAX level was decreased in HPASMCs under hypoxia condition, while si‐circST6GAL1 introduction abolished the effects mediated by hypoxia. In all, circST6GAL1 silencing reversed hypoxia‐induced proliferation and migration, as well as apoptosis arrest in HPASMCs.

### 3.3 MiR‐509‐5p is a target of circST6GAL1

**FIGURE 3:**
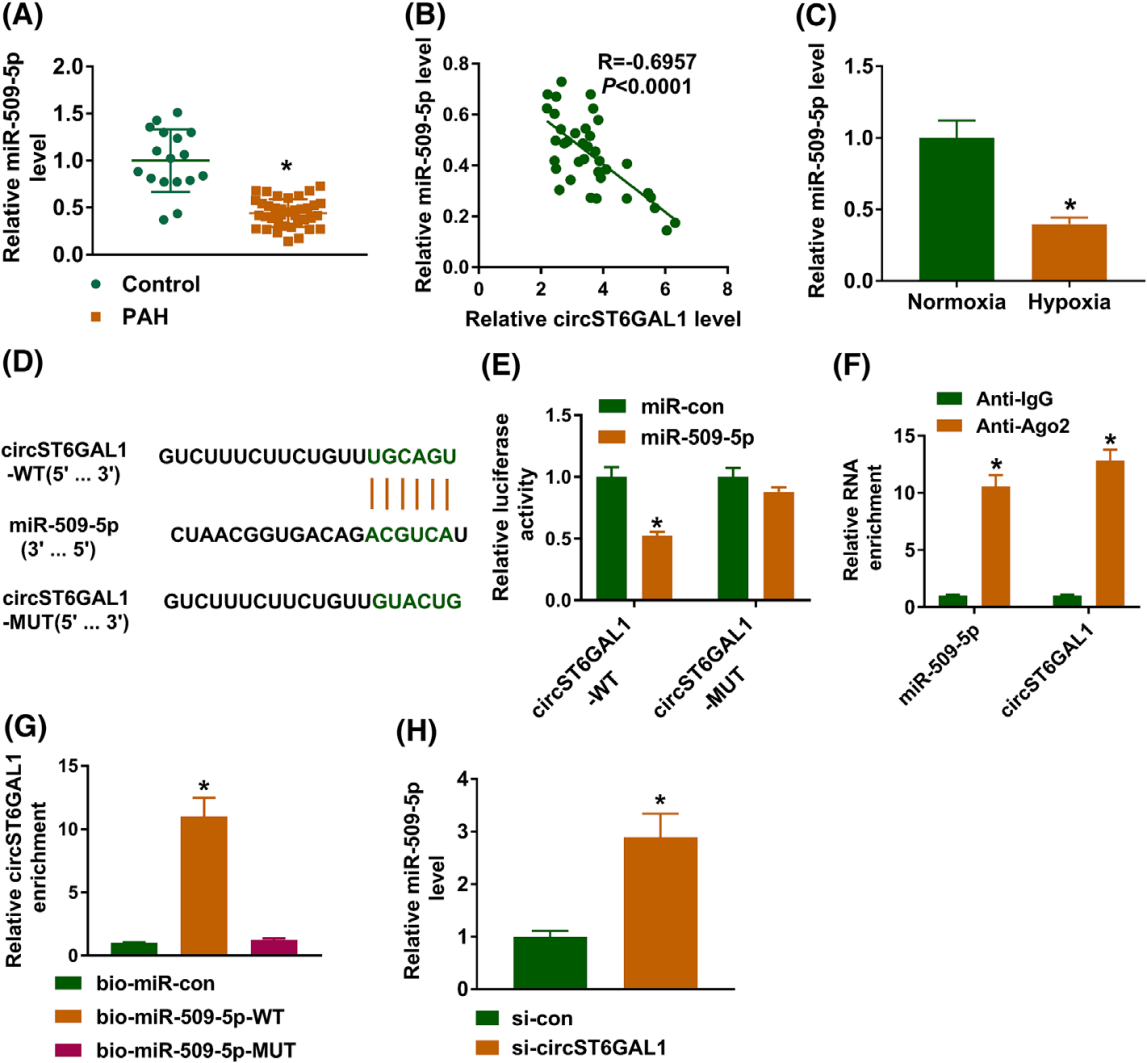
MiR‐509‐5p is a target of circST6GAL1. (A) qRT‐PCR analysis for the expression of miR‐509‐5p in pulmonary arterial hypertension (PAH) patients or healthy individuals. (B) Correlation analysis between circST6GAL1 and miR‐509‐5p expression in PAH patients. (C) qRT‐PCR analysis for miR‐509‐5p expression in hypoxia‐ or normoxia‐induced human pulmonary artery smooth muscle cells. (D) The binding site between miR‐509‐5p and circST6GAL1. (E–G) The interaction between miR‐509‐5p and circST6GAL1 was validated using dual‐luciferase reporter assay, RNA immunoprecipitation assay, and pull‐down assay. (H) qRT‐PCR analysis for miR‐509‐5p expression in human pulmonary artery smooth muscle cells transfected with si‐circST6GAL1 or si‐con. *P < 0.05.

Given the cytoplasmic location of circST6GAL1, circST6GAL1 may function via acting as a sponge for miRNA. The underlying miRNAs of circST6GAL1 were predicted using Circbank database, and six miRNAs (miR‐361‐3p, miR‐509‐5p, miR‐212‐5p, miR‐181a‐5p, miR‐181b‐5p, and miR‐885) that have been reported to be decreased in PAH and show protective effects were chosen. Then, RNA pull‐down assay using circST6GAL1 probes was conducted, and we found that miR‐509‐5p was markedly captured by circST6GAL1 (Figure S1A). Therefore, miR‐509‐5p was selected for subsequent analysis. We observed that miR‐509‐5p was decreased in PAH patients (Figure 3A) and was negatively correlated with circST6GAL1 (Figure 3B). Similarly, a decreased miR‐509‐5p was also found in hypoxia‐induced in HPASMCs (Figure 3C). Then, the interaction between miR‐509‐5p and circST6GAL1 was then explored. The binding site between them was shown in Figure 3D. The dual‐luciferase reporter assay manifested that miR‐509‐5p mimic markedly decreased the luciferase activities in HPASMCs transfected with circST6GAL1‐WT vector, but not in cells with circST6GAL1‐MUT vector (Figure 3E). RIP assay displayed that circST6GAL1 and miR‐509‐5p were markedly pulled down by Ago2 antibody compared with the negative control IgG antibody (Figure 3F). Moreover, pull‐down assay showed circST6GAL1 was notably captured by bio‐miR‐109‐5p‐WT probes (Figure 3G). In addition, circST6GAL1 silencing led to an increase of miR‐509‐5p level in HPASMCs (Figure 3H). Thus, these data confirmed miR‐509‐5p was a target of circST6GAL1.

### 3.4 CircST6GAL1 knockdown reverses hypoxia‐induced HPASMC dysfunction via miR‐509‐5p

**FIGURE 4:**
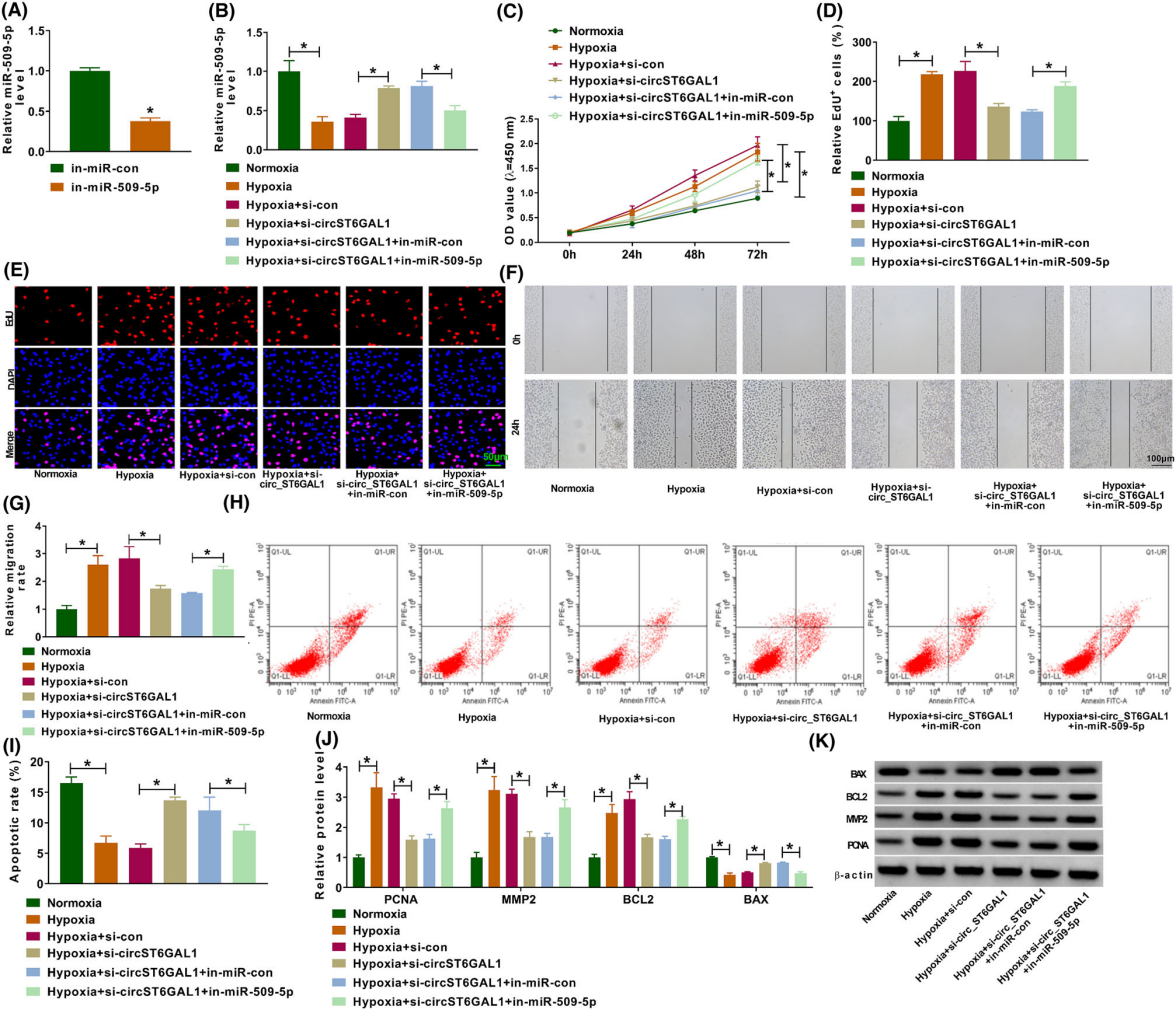
CircST6GAL1 knockdown reverses hypoxia‐induced human pulmonary artery smooth muscle cell dysfunction via miR‐509‐5p. (A) The interference efficiency of in‐miR‐509‐5p or in‐miR‐con was validated using qRT‐PCR. (B–K) Human pulmonary artery smooth muscle cells were transfected with si‐circST6GAL1 alone or si‐circST6GAL1 and in‐miR‐509‐5p, followed by hypoxia stimulation. (B) qRT‐PCR analysis for miR‐509‐5p expression in cells. (C–E) Cell counting kit‐8 and 5‐ethynyl‐2′‐deoxyuridine (EdU) assays for proliferation analysis. (F, G) Wound healing assay for cell migration. (H, I) Flow cytometry for cell apoptosis. (J, K) Western blotting analysis for the protein levels of PCNA, MMP2, BCL2, and BAX. *P < 0.05.

Subsequently, we studied whether circST6GAL1 exerts its effects via miR‐509‐5p. The miR‐509‐5p inhibitor (in‐miR‐509‐5p) was designed, and then, qRT‐PCR analysis showed that in‐miR‐509‐5p introduction markedly reduced miR‐509‐5p expression in HPASMCs (Figure 4A). Thereafter, HPASMCs were transfected with si‐circST6GAL1 alone or si‐circST6GAL1 and in‐miR‐509‐5p, followed by hypoxia stimulation. As expected, si‐circST6GAL1‐induced increase of miR‐509‐5p level in hypoxia‐induced HPASMCs was decreased by in‐miR‐509‐5p (Figure 4B). In further functional analysis, we confirmed that miR‐509‐5p inhibition reversed circST6GAL1 silencing‐evoked arrest of cell proliferation (Figure 4C–E), migration (Figure 4F,G), and promotion of cell apoptosis (Figure 4H,I) in HPASMCs under hypoxia stimulation. Besides, in‐miR‐509‐5p transfection elevated PCNA, MMP2, and BCL2 protein levels and reduced BAX protein level in circST6GAL1‐decreased HPASMCs in the context of hypoxia (Figure 4J,K). Collectively, circST6GAL1 regulated hypoxia‐induced HPASMC dysfunction via miR‐509‐5p.

### 3.5 MCTP2 is a target of miR‐509‐5p

**FIGURE 5:**
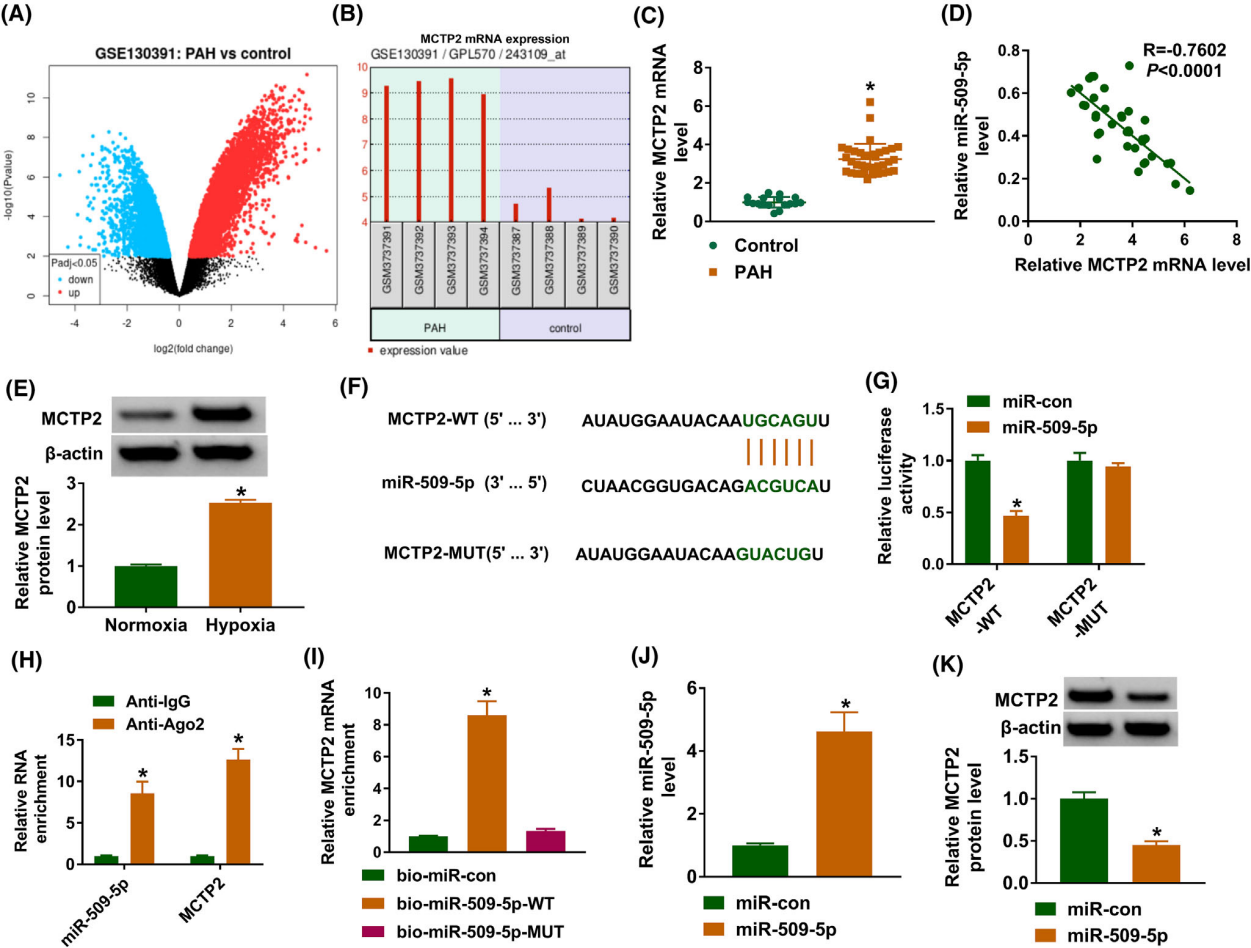
Multiple C2 and transmembrane domain containing 2 (MCTP2) is a target of miR‐509‐5p. (A) The volcano plot visualizes the dysregulated genes between pulmonary arterial hypertension (PAH) samples and healthy control. (B) MCTP2 expression profiles in PAH patients and the control based on GSE130391 dataset. (C) qRT‐PCR analysis for MCTP2 expression in PAH patients or healthy individuals. (D) Correlation analysis between MCTP2 and miR‐509‐5p expression in PAH patients. (E) Western blotting for MCTP2 expression in PAH patients or healthy individuals. (F) The binding site between MCTP2 and miR‐509‐5p. (G–I) The interaction between miR‐509‐5p and MCTP2 was validated using dual‐luciferase reporter assay, RNA immunoprecipitation assay, and pull‐down assay. (J) The elevation efficiency of miR‐509‐5p or miR‐con was validated by qRT‐PCR. (K) Western blotting for MCTP2 expression in human pulmonary artery smooth muscle cells transfected with miR‐509‐5p or miR‐con. *P < 0.05.

Thereafter, the targets of miR‐509‐5p were investigated. According to the prediction of microT_CDS database, many mRNAs were found to have binding sites on miR‐509‐5p. Based on published researches, five genes (DNMT1, MFN1, CDK6, MCTP2, and KLF5) that have been indicated to be elevated in PAH and associated with PAH progression were selected. Through the pull‐down assay, we found that MCTP2 was markedly captured by bio‐miR‐109‐5p probes (Figure S1B). Thus, MCTP2 was selected for subsequent analysis. With a cut‐off criteria of fold change >2.0 and *P* < 0.05, a total of genes were differentially expressed between PAH patients and normal control according to the GSE130391 dataset (Figure 5A). Among which, MCTP2 was significantly upregulated in PAH patients (Figure 5B). Moreover, MCTP2 mRNA level was higher in clinical PAH samples than those in normal control (Figure 5C) and was negatively correlated with miR‐509‐5p (Figure 5D). Also, its protein level was also increased in clinical PAH samples (Figure 5E). The binding site between MCTP2 and miR‐509‐5p was shown in Figure 5F. The dual‐luciferase reporter assay suggested that the luciferase activities were markedly declined in HPASMCs transfected with MCTP2‐WT vector and miR‐509‐5p mimic (Figure 5G). RIP assay showed that MCTP2 and miR‐509‐5p were enriched in Ago2 antibody compared with the negative control IgG antibody (Figure 5H). Moreover, pull‐down assay showed MCTP2 was notably pulled down by bio‐miR‐109‐5p‐WT probes (Figure 5I). After confirming the elevation efficiency of miR‐109‐5p mimic (Figure 5J), Western blotting analysis showed miR‐109‐5p mimic led to a decreased of MCTP2 level in HPASMCs (Figure 5K). Altogether, MCTP2 was a target of miR‐509‐5p.

### 3.6 MiR‐509‐5p reverses hypoxia‐induced HPASMC dysfunction via MCTP2

**FIGURE 6:**
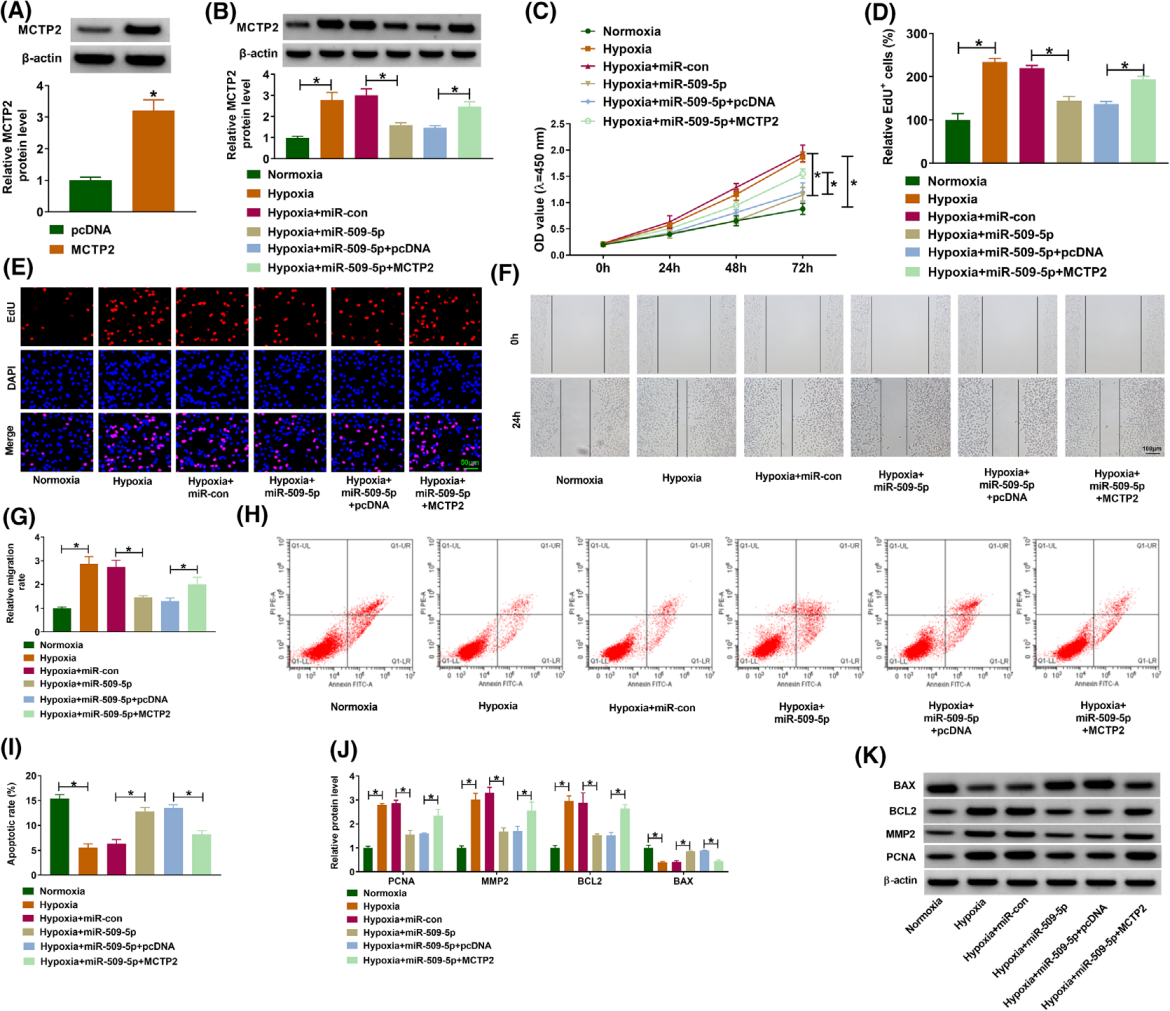
MiR‐509‐5p reverses hypoxia‐induced human pulmonary artery smooth muscle cell dysfunction via multiple C2 and transmembrane domain containing 2 (MCTP2). (A) The elevation efficiency of MCTP2 or pcDNA was validated using Western blotting. (B–K) Human pulmonary artery smooth muscle cells were transfected with miR‐509‐5p alone or miR‐509‐5p and MCTP2, followed by hypoxia stimulation. (B) Western blotting for MCTP2 expression in cells. (C–E) Cell counting kit‐8 and 5‐ethynyl‐2′‐deoxyuridine (EdU) assays for proliferation analysis. (F, G) Wound healing assay for cell migration. (H, I) Flow cytometry for cell apoptosis. (J, K) Western blotting analysis for the protein levels of PCNA, MMP2, BCL2, and BAX. *P < 0.05.

Next, we evaluated the action of miR‐509‐5p/MCTP2 axis on hypoxia‐induced HPASMC dysfunction. MCTP2 overexpression plasmids were designed, and Western blotting analysis showed its introduction markedly elevated MCTP2 expression (Figure 6A). Then, HPASMCs were transfected with miR‐509‐5p alone or miR‐509‐5p and MCTP2, followed by hypoxia stimulation. The introduction of MCTP2 markedly rescued miR‐509‐5p‐induced decrease of MCTP2 level in HPASMCs under hypoxia stimulation (Figure 6B). Functionally, miR‐509‐5p overexpression reversed hypoxia‐evoked promotion on cell proliferation (Figure 6C–E), migration (Figure 6F,G) and inhibition on cell apoptosis (Figure 6H,I) in HPASMCs, while these effects mediated by miR‐509‐5p were abolished after MCTP2 upregulation (Figure 6C–I). In addition, the related markers were determined, and Western blotting manifested that miR‐509‐5p reduced the protein levels of PCNA, MMP2, and BCL2, as well as increased the protein level of BAX in HPASMCs under hypoxia; however, the expression changes caused by miR‐509‐5p were abolished by MCTP2 overexpression (Figure 6J,K). In short, miR‐509‐5p weakened hypoxia‐induced HPASMC dysfunction via MCTP2.

### 3.7 CircST6GAL1 positively regulates MCTP2 via targeting miR‐509‐5p

**FIGURE 7:**
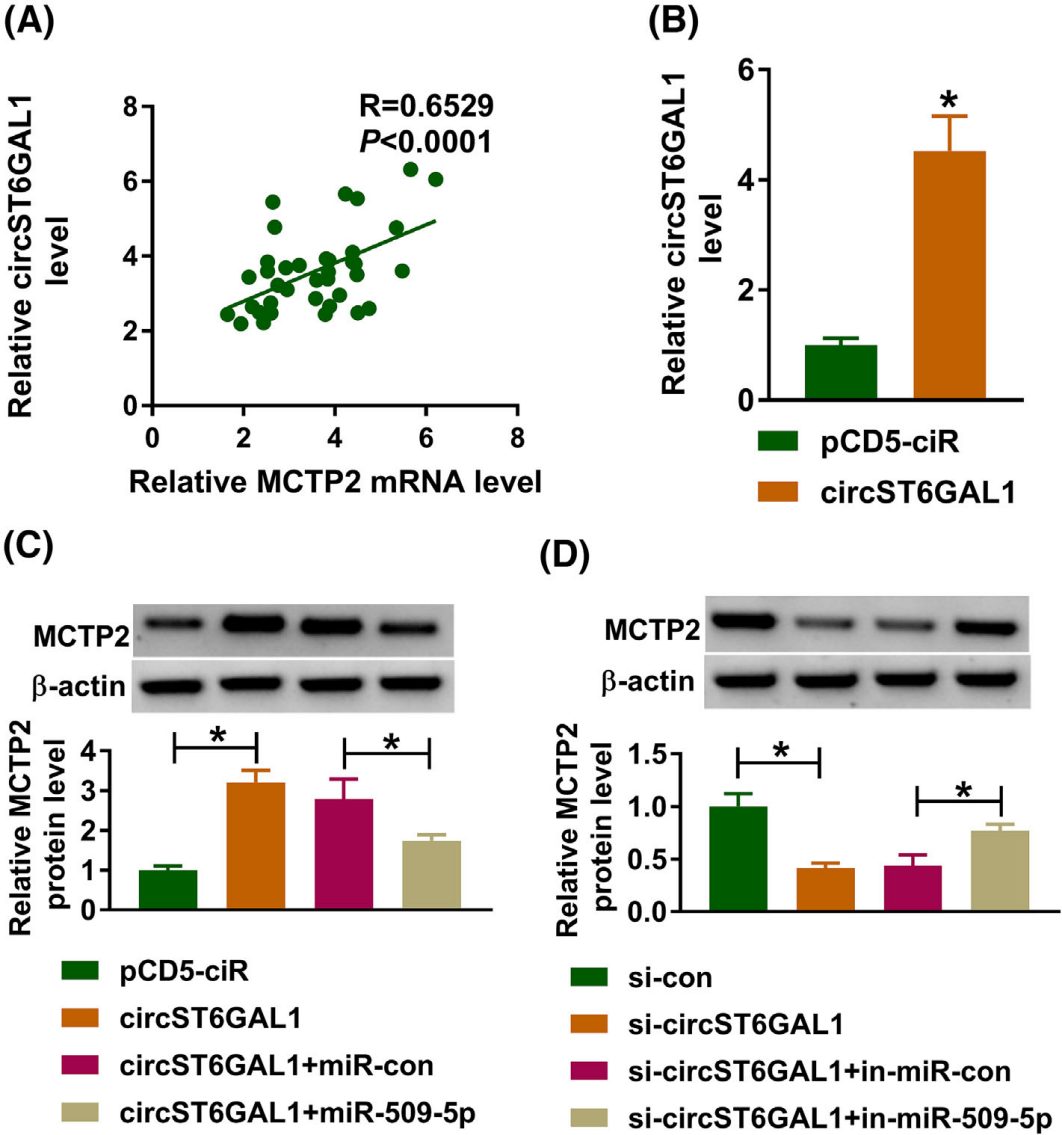
CircST6GAL1 positively regulates multiple C2 and transmembrane domain containing 2 (MCTP2) via targeting miR‐509‐5p. (A) Correlation analysis between MCTP2 and circST6GAL1 expression in pulmonary arterial hypertension patients. (B) The elevation efficiency of circST6GAL1 or pCD5‐ciR was validated using Western blotting. (C, D) The effects of circST6GAL1/miR‐509‐5p axis on MCTP2 expression were determined using Western blotting in human pulmonary artery smooth muscle cells. *P < 0.05.

There was a positive correlation between MCTP2 and circST6GAL1 expression in PAH samples (Figure 7A). CircST6GAL1 overexpression plasmids notably upregulated circST6GAL1 expression in HPASMCs (Figure 7B). Then, we found that circST6GAL1 overexpression induced an increase of MCTP2 expression, which was reduced by miR‐509‐5p overexpression (Figure 7C). Besides, the levels of MCTP2 were decreased after circST6GAL1 silencing and were rescued in response to the introduction of in‐miR‐509‐5p in HPASMCs (Figure 7D).

### 3.8 CircST6GAL1 silencing protects mice from pulmonary artery remodeling and PAH

**FIGURE 8:**
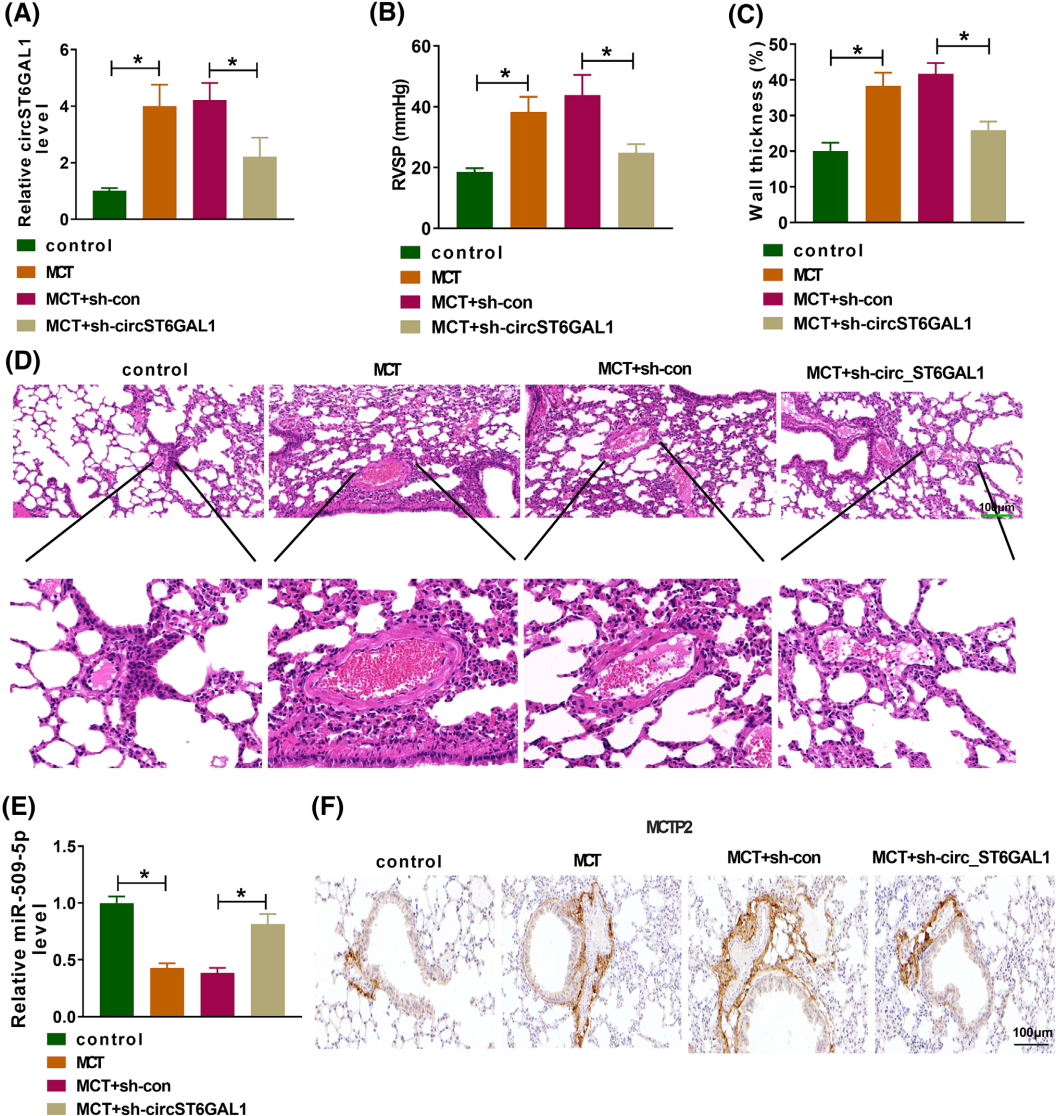
CircST6GAL1 silencing protects mice from pulmonary artery remodeling and pulmonary arterial hypertension. Monocrotaline (MCT)‐induced mice pulmonary arterial hypertension models were established. (A) Pulmonary artery specimens were collected and circST6GAL1 levels were detected by qRT‐PCR. (B) The right ventricular systolic pressure (RVSP) was measured after 3 weeks MCT or saline injection. (C, D) Wall thickness (%) of small pulmonary arteries and hematoxylin and eosin staining for morphological analysis. (E) qRT‐PCR analysis for miR‐509‐5p in pulmonary artery specimens. (F) Immunohistochemistry analysis for MCTP2 protein in pulmonary artery specimens. *P < 0.05.

To probe the action of circST6GAL1 in vivo, MCT‐induced mice PAH models were established. As shown in Figure 8A, circST6GAL1 levels in pulmonary artery specimens of MCT group were higher, while its levels were decreased by circST6GAL1 knockdown. The mean RVSP was markedly higher in MCT group than that in control group, while sh‐circST6GAL1 led to a decrease in mean RVSP compared with the MCT+ sh‐NC group (Figure 8B). In addition, hematoxylin and eosin staining exhibited that the medial walls of the pulmonary small arteries were thickened after circST6GAL1 knockdown in MCT‐induced PAH mice (Figure 8C,D). Thereafter, Western blotting and immunohistochemistry analyses suggested miR‐509‐5p level was decreased, while MCTP2 protein was increased in MCT‐induced PAH, which were attenuated by circST6GAL1 silencing (Figure 8E,F).

## 4 DISCUSSION

Hypertension is a major contributing factor of cardiovascular diseases. Vascular remodeling is a hallmark in the pathophysiology of PAH and characterized by the deposition of extracellular matrix and pulmonary artery medial thickening.^18^ PASMCs are the main cellular components of the medial layer of the pulmonary artery, and VSMCs undergo a phenotypic switch from contractile to synthetic proliferative, dedifferentiated, and migratory phenotypes, accounting for vascular remodeling in PAH.^19^, ^20^ Currently, many circRNAs have been identified to affect VSMC phenotype switching to involve in the progression of hypertension. CircHIPK2 enhanced angiotensin II‐triggered VSMC phenotypic switch via miR‐145‐5p/ADAM axis in hypertension.^21^ CircSirtuin1 suppressed the proliferative, migratory, and autophagic abilities in PASMCs to relieve pulmonary hypertension (PH).^22^ Circ‐myh8 overexpression accelerated PH in chronic hypoxic mice and induced PASMC proliferation in vitro and in vivo.^23^ In our study, an elevated circST6GAL1 expression in the serum of PAH patients and hypoxia‐challenged HPASMCs was observed, indicating the potential implication of circST6GAL1 in PAH. Functionally, silencing of circST6GAL1 counteracted hypoxia‐evoked proliferation, migration, and apoptosis arrest in HPASMCs, implying that circST6GAL1 may promote vascular remodeling by inducing VSMC synthetic switch to affect PAH. Immediately, we confirmed that circST6GAL1 silencing alleviated pulmonary artery remodeling and PAH in MCT‐induced PAH model in vivo. At present, circRNAs have been identified that may serve as promising targets of therapeutic strategies for diseases due to their high stability and the characteristic of tissue‐ or cell type‐specific expression.^24^, ^25^ Moreover, circRNA‐based vaccines, such as CircRNA^RBD^, VFLIP‐X, cytokine‐encoding circular mRNA for cancer therapy, and CircRNA^OVA‐luc^‐LNP vaccine, have been proposed and confirmed in animal models.^26^ Therefore, targeting circST6GAL1 may be a promising strategy for preventing PAH.

MiRNAs have been reported to contribute to the pulmonary artery phenotype of PH.^27^ For example, miR‐17‐5p promoted hypoxia‐evoked proliferation in HPASMCs by regulating p21 and PTEN during PH.^28^ Xu et al. showed that miR‐485‐5p weakened the migration and proliferation of PASMCs in vitro and alleviated PAH in vivo.^29^ MiR‐627‐5p alleviated abnormal migration and proliferation in PASMCs by blocking PI3K/AKT pathway in a MAP 2K4‐dependent manner.^30^ In our work, miR‐509‐5p was verified to be targeted by circST6GAL1. PAH patients' hypoxia‐treated PASMCs showed reduced miR‐509‐5p level, and forced expression of miR‐509‐5p suppressed the migratory and proliferative capacities and induced apoptosis in PASMCs by targeting DNMT1,^31^ which were consistent with our findings. However, our study also confirmed the upstream regulatory factor of miR‐509‐5p in HPASMCs; moreover, circST6GAL1 exerted its regulatory effects in HPASMCs via miR‐509‐5p. In addition, we also confirmed that miR‐509‐5p targeted MCTP2, and circST6GAL1 could positively modulate MCTP2 expression via sponging miR‐509‐5p, suggesting the circST6GAL1/miR‐509‐5p/MCTP2 axis in HPASMCs. According to the GSE130391 dataset, MCTP2 was significantly upregulated in PAH patients. Functionally, MCTP2 overexpression abolished the suppressing effects mediated by miR‐509‐5p on HPASMC migration and proliferation under hypoxia condition.

In all, circST6GAL1 deficiency attenuated the migration and proliferation and induced apoptosis in HPASMCs under hypoxia condition in vitro and alleviated pulmonary artery remodeling and PAH in PAH mouse models in vivo via miR‐509‐5p/MCTP2 axis. These data suggested a molecular theoretical basis for targeted clinical administration in PAH.

## AUTHOR CONTRIBUTIONS

Xing Zhang and Hao Qin conducted the experiments and drafted the manuscript. Qiang Ma and Junbo Zhang collected and analyzed the data. Hongyan Tian operated the software and edited the manuscript. Yan Meng designed and supervised the study. All authors reviewed the manuscript.

## CONFLICT OF INTEREST STATEMENT

The authors declare that they have no conflicts of interest.

## FUNDING INFORMATION

This study was supported by the project fund from Key Research and Development Program of Shaanxi (program no. 2022SF‐402).

## ETHICS APPROVAL AND CONSENT TO PARTICIPATE

This study was authorized by the Ethics Committee of the First Affiliated Hospital of Xi'an Jiaotong University and was carried out according to the guidelines of Declaration of Helsinki, and the written informed consents from the all subjects had been collected. The animal study was approved by the Ethics Committee of the First Affiliated Hospital of Xi'an Jiaotong University. Animal studies were performed in compliance with the ARRIVE guidelines and the Basel Declaration.

## Supporting information


**Figure S1.** **The selection of target miRNAs or mRNAs.** (A, B) RNA pull‐down assay using the circST6GAL1 or miR‐509‐5p probes was performed to select the targets. *P < 0.05.

## Data Availability

The datasets used and analyzed during the current study are available from the corresponding author on reasonable request.
