# Supplementary figures and images for: CircST6GAL1 knockdown alleviates pulmonary arterial hypertension by regulating miR‐509‐5p/multiple C2 and transmembrane domain containing 2 axis
Source: Clin Respir J. 2024 May 15;18(5):e13771. doi: 10.1111/crj.13771 (PMC11094577; doi:10.1111/crj.13771)

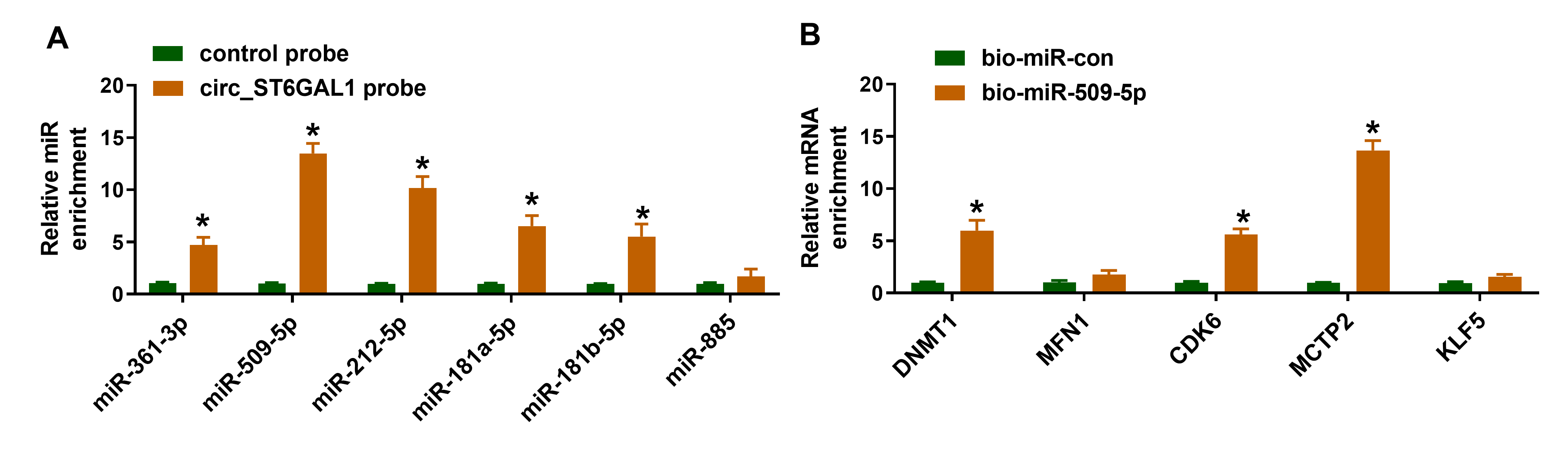

Supplement: Supplementary file 1 — Figure S1. The selection of target miRNAs or mRNAs. (A, B) RNA pull‐down assay using the circST6GAL1 or miR‐509‐5p probes was performed to select the targets. *P < 0.05. [file CRJ-18-e13771-s001.tif]
